# Prehospital hyperoxemia does not influence the functional neurological outcome in polytraumatized patients with traumatic head injury

**DOI:** 10.1186/cc12223

**Published:** 2013-03-19

**Authors:** V Vujanovic Popovic, T Pelcl, M Spindler, Z Klemenc Ketis, M Strnad

**Affiliations:** 1 ZD dr. Adolfa Drolca Maribor, Slovenia; 2 University of Maribor, Slovenia

## Introduction

The association between hyperoxemia and neurological outcome in trauma patients is not clear. We examined the association between prehospital hyperoxemia and neurological outcome in polytraumatized patients.

## Methods

This was a retrospective study of polytraumatized patients with traumatic head injury who were endotracheal intubated and ventilated with supplemental oxygen (100%) in the prehospital emergency setting. Arterial partial oxygen pressure (PaO_2_) was measured after arrival at the hospital trauma center. We included patients with initial PaO_2 _above 160 mmHg (hyperoxemia group). The severity of the trauma was determined upon the admission to the hospital by the Injury Severity Scale (ISS) and the outcome was assessed at the discharge from the hospital using the Glasgow Coma Scale (GCS), Glasgow Outcome Scale (GOS) and Cerebral Performance Categories scale (CPC). Mann-Whitney's test was used for data analysis.

## Results

Sixty patients were involved in the study. Forty-eight (80%) of them were men and 86.7% sustained blunt trauma. Hyperoxemia was present in 41.6% of patients. Initial average ISS was 38, in patients with normoxemia 32.5 and in patients with hyperoxemia 35.4. Discharge GCS, GOS and CPC in the hyperoxemia group compared with the normoxemia group were 9.86 versus 9.33 (*P *= 0.503), 2.52 versus 2.24 (*P *= 0.613) and 3.10 versus 3.19 (*P *= 0.936) with the duration of hospitalization of 26.64 days versus 27.72 days (*P *= 0.984).

## Conclusion

Prehospital hyperoxemia did not influence the functional neurological outcome. One of the reasons for this finding could be the short arrival time to the trauma center where repeated analyses of arterial blood gases were performed. Therefore, correction of fraction of inspired oxygen according to the arterial blood gas analysis shortens the time of hyperoxemia, thus reducing neuronal brain damage.
